# Advancing the Study of Loneliness and Health in Older Adults Through the Use of Integrative Data Analysis

**DOI:** 10.1093/geroni/igab046.818

**Published:** 2021-12-17

**Authors:** Anthony Ong, Eileen Graham, Kathryn Jackson, Emorie Beck, Jing Luo, Olivia Atherton, Emily Willroth, Daniel Mroczek

**Affiliations:** 1 Cornell University, Ithaca, New York, United States; 2 Northwestern University, Chicago, Illinois, United States; 3 Northwestern university, Chicago, Illinois, United States; 4 Northwestern University Feinberg School of Medicine, Chicago, Illinois, United States

## Abstract

Recent work has shown the importance of studying loneliness and social isolation across adulthood for understanding healthy aging. This project explored loneliness trajectories across multiple independent samples. Using coordinated IDA, we estimated and meta-analyzed identical multilevel growth models in loneliness using three samples (ELSA, SHARE, HRS). We found u-shaped change, suggesting that loneliness may decline from young adulthood to midlife, then increase after midlife. These trajectories were significant across all three datasets and not fully explained by demographics or depression. We found that divorce, widowhood, social isolation, and functional limitations were associated with higher overall loneliness. Additionally, divorce and functional limitations, and sex (being male) were associated with deeper dips in loneliness in midlife and steeper increases in old age. These findings suggest that loneliness increases across the second half of life and point to the need for evidence-based strategies for addressing social disparities in midlife and later adulthood.

